# The hUC-MSCs cell line CCRC-1 represents a novel, safe and high-yielding HDCs for the production of human viral vaccines

**DOI:** 10.1038/s41598-017-11997-1

**Published:** 2017-10-02

**Authors:** Ping Chen, Ke-Hua Zhang, Tao Na, Lin Wang, Wei-Dong Yin, Bao-Zhu Yuan, Jun-Zhi Wang

**Affiliations:** 1 Cell Collection and Research Center, National Institutes for Food and Drug Control, Beijing, 100050 PR China; 2 Department of Oncology, the Affiliated Hospital of Southwest Medical University, Luzhou, P.R. China; 3 Sino Vac Biotech, Beijing, 100085 PR China

## Abstract

MRC-5 represents the most frequent human diploid cells (HDCs)-type cell substrate in the production of human viral vaccines. However, early-passage MRC-5 is diminishing and, due to both technical and ethical issues, it is extremely difficult to derive novel HDCs from fetal lung tissues, which are the common sources of HDCs. Our previous studies suggested that human umbilical cord may represent an alternative but convenient source of new HDCs. Here, we established a three-tiered cell banking system of a hUC-MSC line, designated previously as Cell Collection and Research Center-1 (CCRC-1). The full characterization indicated that the banked CCRC-1 cells were free from adventitious agents and remained non-tumorigenic. The CCRC-1 cells sustained its rapid proliferation even at passage 30 and were susceptible to the infection of a wide spectrum of viruses. Interestingly, the CCRC-1 cells showed much higher production of EV71 or Rubella viruses than MRC-5 and Vero cells when growing in serum-free medium. More importantly, the EV71 vaccine produced from CCRC-1 cells induced immunogenicity while eliciting no detectable toxicities in the tested mice. Collectively, these studies further supported that CCRC-1, and likely other hUC-MSCs as well, may serve as novel, safe and high-yielding HDCs for the production of human viral vaccines.

## Introduction

Cell substrates have been commonly utilized as the most critical starting materials in manufacturing biological products, including both recombinant proteins and vaccines^1,2^. In the production of viral vaccines, different cell substrates may determine a dramatic difference in reactogenicity of manufacturing process, yield of infectious units or antigens, or final preparation^3,4^. In addition, different cell substrates are also associated with the variations in the efficiency of final product purification, especially the removal of residual cellular constituents. Therefore, cell substrates have been viewed as one of the most important starting materials in determining the productivity, stability and quality of the resultant biological products^1–5^.

The cell substrates used in the production of licensed or investigational viral vaccines for human use include primary cells, continuous cell lines (CCLs), and human diploid cell lines (HDCs)^3,6^. Primary cells, such as chicken embryo fibroblasts, hamster kidney cells, are the cells isolated freshly from animal tissues and continue to be employed as the cell substrates for the production of viral vaccines^6,7^. But, they are frequently associated with batch-to-batch variations and high risk of introducing exogenous agents into the cultures and resultant vaccines^4^. CCLs are immortalized cells and provide logistical advantages over primary cell substrates. However, many CCLs often exhibit a variable degree of tumorigenicity, thus often requiring much stringent removal process to strictly control the level of cell substrate residues, such as residual proteins or DNAs, in the final vaccines produced from CCLs^8,9^. HDCs, such as WI-38 and MRC-5, derived from human fetal lungs, maintain normal karyotype as well as non-tumourigenic characteristics during a finite serial propagation. They have been used in the manufacture of human vaccines for many years without causing serious vaccine-associated adverse events and are thus considered as the safest cell substrates for the production of human viral vaccines^10,11^. However, because of the limited propagation capacity as well as ethical issues, continuous supply of low-passage HDCs has always being a critical problem in the field of vaccine production^3^.

Mesenchymal stem cells (MSCs) are a group of fibroblast-like cells with abilities to self-renew and to differentiate into multiple cell lineages, such as osteocytes, chondrocytes and adipocytes^12,13^. A unique feature of MSCs in the focus of recent studies is its unique immunomodulatory activities, which have been implicated in the treatment or prevention of various inflammatory and autoimmune diseases^14–16^. However, developing MSCs as novel cell substrates for the production of viral vaccines has rarely been explored.

Interestingly, our recent studies demonstrated that many HDCs established from fetal lungs, such as MRC-5, exhibited several critical properties of human umbilical cord-derived mesenchymal stem cells (hUC-MSCs), including cell morphology, growth activity, expression of cell surface markers, abilities to differentiate into multiple cell lineages and immunomodulatory activities^17^. In the meantime, it was found that the (Cell Collection and Research Center-1) cells, an hUC-MSC cell line reported in the previous studies, sustained primitive characteristics during extensive expansion and exhibited a similar sensitivity to the infection of EV71 and Rubella viruses as MRC-5, thus suggesting that hUC-MSCs may meet the same requirements as the traditional HDCs for the production of human vaccines^17^.

In the present study, to further develop CCRC-1 as a novel HDC for the production of human vaccines, we first established a three-tiered banking system for CCRC-1, intensely characterized the banked cells for growth activities and tumorigenicity, and then evaluated the susceptibility of the cells to a wide spectrum of viruses and the growth and propagation of both EV71 and Rubella viruses in the cells. With a greater focus on EV71, we also compared the immunogenicity and safety of EV71 vaccines produced in CCRC-1 cells with that from MRC-5 and Vero cells. Finally, we demonstrated that different strains of hUC-MSCs exhibited a similar susceptibility to both EV71 and Rubella infections, therefore concluding that CCRC-1, and perhaps other hUC-MSC cell lines as well, may be used as novel HDCs for the production of human viral vaccines.

## Materials and Methods

### Materials

#### Cells

MRC-5, Vero and RK-13 cells were obtained from the American Type Culture Collection (Rockville, MD, USA), all hUC-MSCs were isolated from Wharton’s jelly of human umbilical cord and fully characterized^17^. MRC-5 was cultured in MEM supplemented with 10% FBS (Gibco, Grand Island, NY, USA), Vero and RK-13 were cultured in MEM supplemented with 10% NBS (Sijiqing, Zhejiang, China), hUC-MSCs were cultured in α-MEM supplemented with 10% FBS.

#### Virus

EV71 strain 523-07 T (EV71/523-07T), Sendai virus (SEV), Adenovirus type 5 (ADV-5) and Herpes simplex virus 2 (HSV-2) were all derived from our institute; EV71 vaccine strain SH06 (EV71/SH06) and Rubella vaccine strain RA27/3 (RUV/RA27/3) were provided by Sinovac Biotech Ltd (Beijing, China); Measles vaccine strain Chang-47 (MV/Chang-47) and Varicella Zoster virus vaccine strain Oka (VZV/Oka) were from Changchun Institute of Biological Products (Changchun, China); Japanese encephalitis vaccine strain SA14-14-2 (JEV/SA14-14-2) was from Chengdu Institute of Biological Products (Chengdu, China).

#### Animals

Suckling mice, adult mice, guinea pigs, rabbits and female nude mice were purchased from the Laboratory Animal Center of National Institutes for Food and Drug Control (NIFDC) (Beijing, China). All studies involving animals were conducted in conformity with institutional guidelines concerning animal use and care and the relevant protocols approved by the NIFDC Institutional Animal Care and Use Committee were followed.

### Generation and characterization of primary, master and working cell banks

The CCRC-1 cells at passage 5 following the initial isolation and proliferation were used as cell seeds, which were expanded in 175-cm^2^ cell culture flasks through consecutive passages to generate a primary cell bank (PCB). Following an industry standard procedure for three-tiered cell banking^1,2^, a master cell bank (MCB) and a working cell bank (WCB) were sequentially generated using the cells from the PCB. A portion of the cells from each bank were intensely evaluated for their freedom from contamination of mycoplasma, bacteria, fungi and viruses according to the recommendations of World Health Organization (WHO) Guidelines as well as the requirements from Chinese Pharmacopeia^18,19^. Briefly, the presence of bacteria and fungi in CCRC-1 cells were tested using the culture method^19^. The mycoplasma test was conducted using both the culture method and indicator cell inoculation method^19^. Unspecified viral agents in general were tested *in vitro* using Vero, MRC-5 and 2BS cells as indicator cells, and *in vivo* through inoculation into embryonated eggs, suckling mice, adult mice, guinea pigs and rabbits^19^. The specific viruses of human, bovine and porcine origins were tested using either PCR-based or indicator cell-based methods^19^. The retrovirus was tested using the enhanced reverse transcriptase assay and transmission electron microscopy for detecting viral particles^19^.

### Tumor formation assay

The tumorigenicity of CCRC-1 cells at different passage levels was evaluated by their abilities to form tumors in adult nude mice. Briefly, the nude mice were injected subcutaneously at the dorsal medial sites with 1 × 10^7^ cells suspended in 200 μl serum-free medium. The injection with 1 × 10^6^ Hela cells suspended in 200 μl of MEM basal medium served as positive control. During a 3-month observation period, the animals were examined for any abnormalities in appearance and behavior, and palpated twice a week to detect nodule formation at the site of inoculation. At the end of the experiment, the animals were euthanized and necropsied. The tissues of the skin at the injection site, and the organs of the adjacent lymph nodes, heart, liver, spleen, lungs, kidney, and any gross lesions were collected. They were then fixed in 10% neutral buffered formalin, embedded in paraffin, sectioned, stained and finally examined under a microscope by a certified pathologist.

### hTERT activity assay

The hTERT activity in CCRC-1 cells at different passage levels and in A549 human lung cancer cells was determined by using TRAPeze XL Telomerase Detection Kit (Millipore, Billerica, MA, USA) according to the procedures described in the manufacturer’s instruction. Briefly, PCR amplification was performed and the products were transferred to 96-well plates. The intensity of the fluorescent signal emitted by the PCR products was measured with Gemini XPS Microplate Reader (Molecular Devices, USA) and used to determine the hTERT activity^17^.

### Cell proliferation assay

The cell proliferation assay starting with 2.5 × 10^5^ cells in T25 cell culture flasks supplied with complete medium was performed by counting cell numbers once a day during an eight day growth period.

### Viral susceptibility test

To determine the viral susceptibility profile of each cell substrate, the CCRC-1, MRC-5 or Vero cells growing to full confluence in T25 cell culture flasks in each complete medium were shifted to MEM supplemented with 2% FBS and then infected with EV71/SH06, MV/Chang-47, RUV/RA27/3, JEV/SA14-14-2, VZV/Oka, SEV, ADV-5 or HSV-2 at an MOI (multiplicities of infection) of 0.1. The cells infected with RUV/RA27/3 were incubated at 32 °C and the cells infected with all other viruses were incubated at 35 °C. After incubation for 6 days, the cytopathic effect (CPE) induced by each viral infection was employed as a measure of the susceptibility to each relevant virus.

### Virus propagation activity

The virus propagation activity was employed to demonstrate the potential of viral vaccine production by each cell substrate and represented commonly by viral content and/or viral titer. For measuring viral replication efficiency in CCRC-1 and MRC-5, equal number of each cells growing to full confluence in T25 flasks were shifted to maintaining in MEM supplemented with 2% FBS, and then were exposed to viruses at MOIs ranging from 0.001 to 0.1, which were equivalent to approximately 2.0 × 10^3^ to 2.0 × 10^5^ CCID50 per 2.0 × 10^6^ cells in 10 ml maintaining media. To determine the viral production in hUC-MSCs derived from different donors, the cells growing to full confluence in T25 cell culture flasks in complete medium were changed to maintain in 10 ml MEM supplemented with 2% FBS and then infected with viruses at MOI 0.1. The EV71/SH06-infected cells were incubated at 35 °C and the RUV/RA27/3-infected cells were incubated at 32 °C. Following the incubation for 6 days, the cells were constantly monitored for the appearance of CPEs and were sampled every 24 h for measuring viral titer or viral antigen.

For vaccine preparation, the cells growing to full confluence in T175 flasks were washed and replaced with serum-free MEM medium. Then, EV71/SH06 virus or RUV/RA27/3 virus was added to each flask at MOIs ranging from 0.001 to 0.1. At day 6 after virus inoculation, the relevant viral vaccines were prepared by collecting the supernatant of the infected cells as followed by a repeated quick freezing-thawing procedure. Before vaccination, the EV71 vaccines were inactivated by incubating the collected supernatant at 56 °C for 30 min.

The virus titer was quantified using the standard CCID50 assay^17^. Briefly, 1 × 10^4^ Vero cells or RK-13 cells were seeded to each well of a 96-well plate, then 100 μl of either EV71 or RUV virus from 10-fold serial dilution was added to each well, and then incubated at 35 °C for 7 days or at 32 °C for 14 days, respectively. At the end of the incubation, the appearance of CPE in over 90% of the infected cells was used to determine viral titer expressed as lgCCID_50_/ml.

A quantitative ELISA assay kit (Sinovac Biotech Ltd, Beijing) was used to detect EV71 antigen content^17^. The reference EV71 standard (1600U/ml, from National Institutes for Food and Drug Control, Beijing) and testing samples were serially diluted in a two-fold manner and tested in duplicate wells. The parallel-line method was used to calculate the antigen content of the samples. Results were expressed in standard national EV71 antigen units/ml (U/ml).

### Evaluation of immunogenicity and toxicity induced by the EV71 vaccines

The immunogenicity elicited by the EV71 vaccines was evaluated by the generation of the EV71 neutralizing antibody in BALB/c mice following the vaccine inoculation. Before testing the neutralizing antibody, the 6–8 week old female mice were randomly divided into 8 groups with 10 mice in each group and intraperitoneally (i.p.) inoculated with: a. PBS; b. 0.5 mg aluminum hydroxide in PBS (Al); c. 500 U of the inactivated Vero cell-derived EV71 viral antigen (Vero); d. 500 U of the inactivated Vero cell-derived EV71 viral antigen absorbed to 0.5 mg of Al (Vero + Al); e. 500 U of the inactivated MRC-5 cell-derived EV71 viral antigen (MRC-5); f. 500 U of the inactivated MRC-5 cell-derived EV71 viral antigen absorbed to 0.5 mg of Al (MRC-5 + Al); g. 500 U of the inactivated CCRC-1 cell-derived EV71 viral antigen (CCRC-1); h. 500 U of the inactivated CCRC-1 cell-derived EV71 viral antigen absorbed to 0.5 mg of Al (CCRC-1 + Al). The sera were collected at both 14 days and 28 days post inoculation, and then inactivated at 56 ± 0.5 °C for 30 min.

The titers of the EV71 neutralizing antibodies (NAbs) in the collected sera were measured by its ability to protect CPE in Vero cells following the method reported previously^20^. Briefly, 50 μl of each diluted serum with the dilution ranging from 1:8 to 1:1024 was mixed with 50 μl EV71/523-07 T of 100 CCID50 per well in a 96-well microplate and incubated at 37 ± 0.5 °C for 2 h. Next, 1.0 × 10^4^ Vero cells suspended in 100 μl MEM complete medium were added to each well. The plate was then incubated at 35 °C ± 0.5 for 7 days and the appearance of CPE in Vero was detected during the incubation period. The NAbs titer of each sample was defined as the highest sera dilution that was capable of inhibiting 50% of CPE. The NAbs titer lower than 1:8 dilution were assigned as value 1:4, while the NAbs titer equal to or above 1:1536 dilution was assigned as value 1:1536.

To investigate the toxicities associated with the vaccine inoculation, the animals following the inoculation were evaluated continuously for main toxicity indexes, such as weight loss, fur ruffling, abnormal behavior, diarrhea, anorexia, skin ulceration, and toxic deaths. At the end of observation, the mice were euthanized by cervical dislocation and the major tissues or organs of each mouse were biopsied and analyzed for any pathological findings.

### Statistical analysis

Statistical analyses were performed using SPSS version 17.0. Results were presented as mean ± standard deviation (SD) of the data from three independent experiments. For statistical comparison between groups, Student’s *t* test was used, with a *p* value less than 0.05 considered significant.

## Results

### Generation and characterization of CCRC-1 cell banks

Following our previous study, to further validate that hUC-MSCs can potentially serve as a novel group of HDCs, and to establish CCRC-1 as a novel HDC cell substrate for vaccine production, the CCRC-1 cells cultured in α-MEM complete medium was continuously expanded by observing an industry cell banking standard to generate a three-tiered cell banking system, in which the PCB was at passage 9, the MCB at passage 13, and the WCB at passage 17^1–4^. By following the requirements of the relevant bank characterization guidelines^18,19^, the cell banks were fully characterized for their freedom from contamination with a spectrum of adventitious agents. The bank characterization was performed by utilizing both *in vitro* and *in vivo* tests, including the inoculation of the testing materials into indicator cells, specific pathogen free (SPF) embryonated eggs, mice, guinea pigs, or rabbits (Table 1). As summarized in Table 1, the three-tiered banks showed freedom from contamination by bacteria, fungus, mycoplasmas, species-specific viruses, retroviruses or any unspecified adventitious agents.

**Table 1:** The CCRC-1 cell bank characterization for testing adventitious agents.

Cell banks	Test Entities	Methods	Result
PCB, MCB and WCB	Bacterial and fungi	Sterility test*	Negative
	Mycoplasmas	Culture method*	Negative
		Indicator cell inoculation and staining*	Negative
	Viruses	*In vitro* tests using indicator cells*	Negative
		Nucleic acid testing for species-specific viruses	
		*In vivo* animal inoculation tests using the embryonated eggs, suckling mice, adult mice and rabbit and guinea pigs	Negative
		Transmission Electron Microscopy test for viral particles	Negative
		Tests for retroviruses*	

In the cell bank characterization, the MCB (master cell bank) was comprehensively tested for all testing items by all testing methods, whereas the PCB (primary cell bank) and WCB (working cell bank) were tested only by the *-labeled test methods.

### CCRC-1 cells maintain non-tumorigenic feature and a high growth activity during the extended proliferation

To meet the requirements of non-tumorigenicity for HDCs^2^, the CCRC-1 cells at various passage levels were evaluated using nude mice tumor formation assay. As a result, while the Hela cells, used as a positive control in the assay, readily showed a progressive tumor formation starting at the first 30 day after inoculation in all animals tested, no palpable nodules could be detected in the mice inoculated with CCRC-1 cells at passage levels of P5, P15 and P30 during the entire observation period. In addition, the biopsied tissues showed no existence of tumor cells under microscopic examination in all test groups (Table 2).

**Table 2:** Numbers of animals injected with either Hela or CCRC-1 cells and the corresponding tumor incidence.

Groups	Number of nude mice injected (female)	Nodule at the injection site	Tumors beyond the inoculation sites
Hela cells	10	10	0
CCRC-1 (P5)	10	0	0
CCRC-1 (P15)	10	0	0
CCRC-1 (P30)	10	0	0

**Figure 1:**
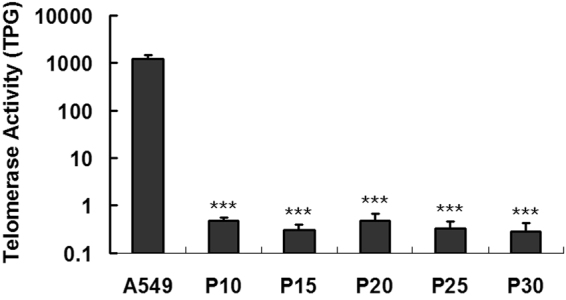
The hTERT activity of CCRC-1 cells. A TRAPeze XL Telomerase Detection Kit was used to detect the hTERT activity of CCRC-1 cells at passage 10, 15, 20, 25 and 30. A549 human lung cancer cells were used as positive control. The hTERT activities are expressed as Total Product Generated (TPG). Data are represented as the mean ± SD of three independent experiments. (***P < 0.001, compared with A549).

To further exclude the tumorigenic transformation of CCRC-1 cells, we also evaluated the hTERT activity of the cells at passage levels of 10, 15, 20, 25 and 30. The results showed that, while the control A549 lung cancer cells exhibited approximately 1200 TPG of hTERT activity, the CCRC-1 cells at all passages exhibited an extremely low hTERT activity (Fig. 1).

**Figure 2:**
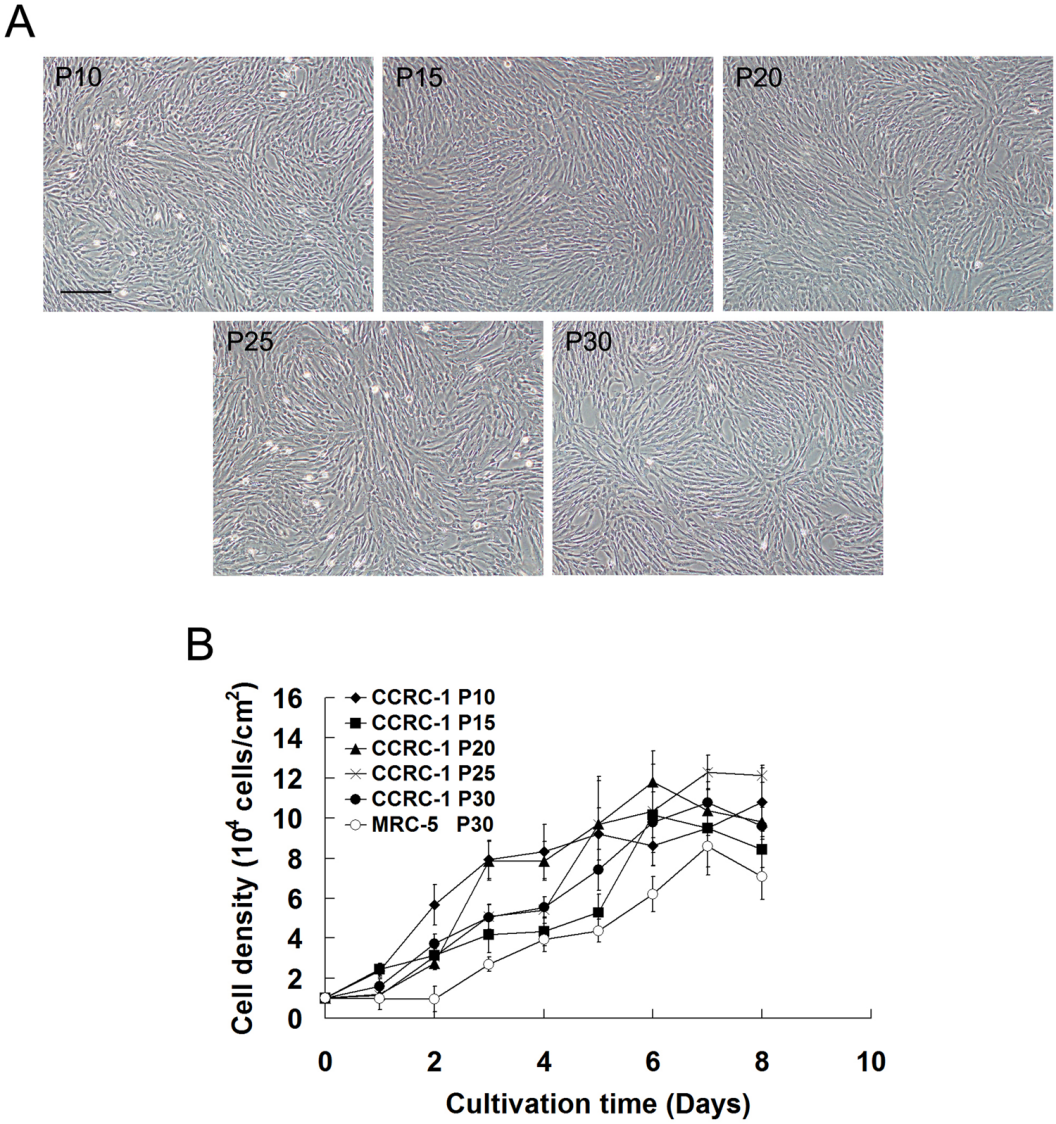
Growth parameters of CCRC-1 cells. The exponential cell growth was observed during the first 8 days post-seeding. (**A**) Representative images of the morphology of CCRC-1 cells at passage 10, 15, 20, 25 and 30; scale bar = 100 µm. (**B**) Relative growth rates of CCRC-1 cells at different passage levels (10, 15, 20, 25 and 30) were compared with MRC-5 cells at passage 30. The growth activities of the cells are expressed as the cell density at indicated time points. Data are represented as the mean ± SD of three independent experiments.

To determine whether the extended proliferation of CCRC-1 could reduce its growth activity, the cells at different passage levels were tested for their growth pattern and doubling time, the parameters commonly used to indicate the proliferation potential of HDCs^2^. As shown in Fig. 2, the CCRC-1 cells at different passage levels during continuous growth for 8 days did not show a significant difference in both cell morphology and proliferation rate. In addition, it was observed that the peak cell densities of CCRC-1 and MRC-5 cells at passage 30 were (1.08 ± 0.16) × 10^5^ cells/cm^2^ and (0.86 ± 0.10) × 10^5^ cells/cm^2^ (Fig. 2), respectively, thus suggesting that the CCRC-1 cells could reach an even higher peak density than MRC-5 cells at the same passage level (*P* < 0.05). By comparing the population doubling times of CCRC-1 and MRC-5 cells, it was further confirmed that CCRC-1 cells replicated more rapidly than MRC-5 cells at the same passage level (Table 3).

**Table 3:** Population doubling times of the CCRC-1 and MRC-5 cells.

Cells	Population doubling time (h)
CCRC-1 (P10)	30.8 ± 6.2
CCRC-1 (P15)	36.5 ± 4.0
CCRC-1 (P20)	42.8 ± 2.3
CCRC-1 (P25)	41.8 ± 4.3
CCRC-1 (P30)	42.4 ± 3.4
MRC-5 (P30)	48.5 ± 5.4

### The CCRC-1 cells were susceptible to infection of a wide spectrum of viruses

**Figure 3:**
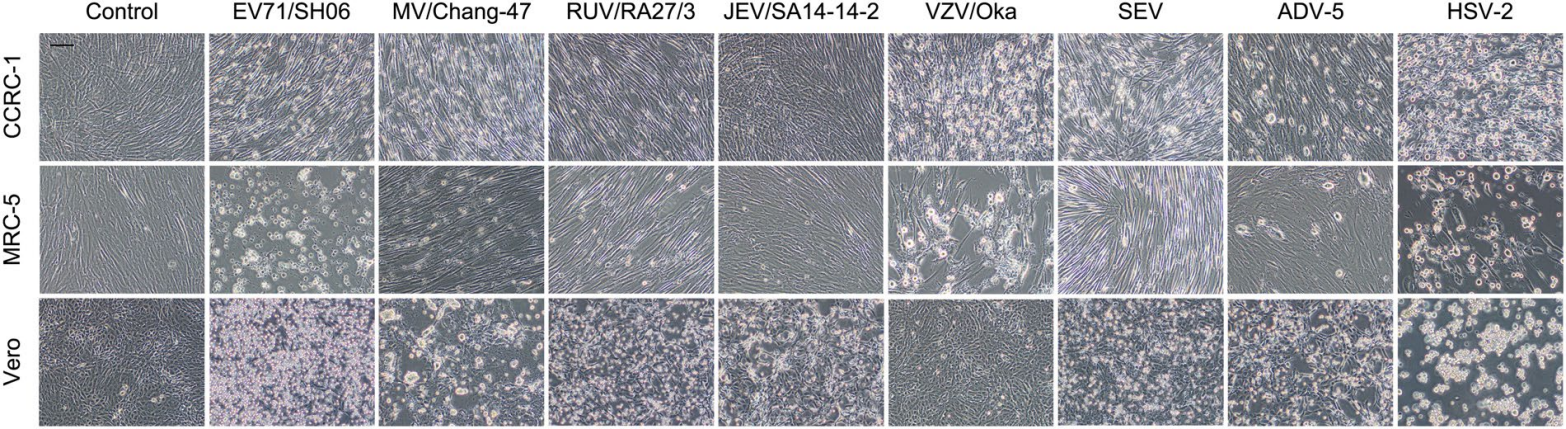
Susceptibility of CCRC-1, MRC-5 and Vero cells to viral infection. The susceptibility of viral infection was determined by the appearance of CPE following the infection of EV71/SH06, MV/Chang-47, RUV/RA27/3, JEV/SA14-14-2, VZV/Oka, SEV, ADV-5 and HSV-2 at 0.1 MOI. Un-infected cells served as the control. Representative images of the morphology taken at day 6 post-infection; scale bar = 100 µm.

Both MRC-5 and Vero cells are highly permissive to infection of various viruses and are commonly used in production of different viral vaccines. To compare the permissiveness of CCRC-1 to viral infections in parallel with MRC-5 and Vero, all three cell lines were infected with various viruses for 6 days. As determined by the appearance of CPE, it was seen that CCRC-1 cells exhibited a similar profile of viral permissiveness as MRC-5 cells with both showing the susceptibility to the infection of EV71/SH06, MV/Chang-47, RUV/RA27/3, VZV/Oka, SEV, ADV-5 and HSV-2. No apparent CPE was induced by JEV/SA14-14-2 in both CCRC-1 and MRC-5 cells. Meanwhile, Vero cells exhibited a significant susceptibility to all infections but not VZV/Oka (Fig. 3).

### CCRC-1 cells are permissive to high viral replication

**Figure 4:**
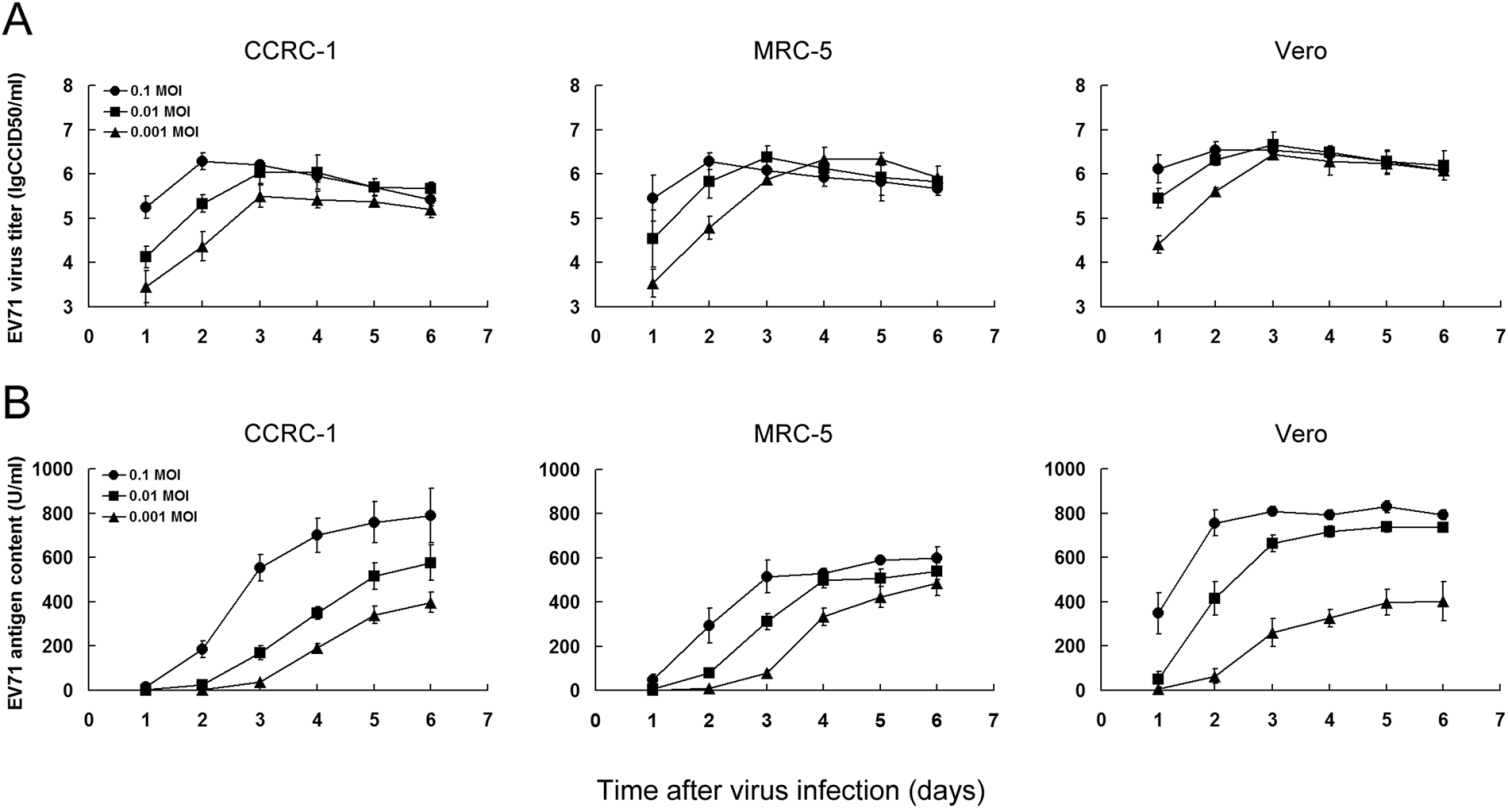
Replication activity of EV71 virus in CCRC-1, MRC-5 and Vero cells. The cells were infected with EV71/SH06 virus at MOIs of 0.1, 0.01 and 0.001, respectively. The virus titers and antigen contents were tested every 24 h post infection for 6 days. (**A**) The replication kinetics are shown in viral titers in the cells. The viral titers were determined using the standard CCID50 assay and expressed as lgCCID50/ml. (**B**) Growth kinetics of viral antigens in the cells. The viral antigens were detected using a quantitative ELISA assay kit and expressed as units/ml (U/ml). Data are shown as the mean ± SD of three independent experiments.

Using EV71 and Rubella viruses as two representatives, we further determined whether the CCRC-1 cells are permissive to high viral replication for evaluating its ability to achieve high efficiency in manufacturing viral vaccines^17^. We first compared CCRC-1 with MRC-5 or Vero for the replication activity of EV71/SH06 virus, which has been utilized as a viral strain in the production of the inactivated EV71 vaccine using Vero cells^20^. It was observed that, as indicated by viral titer, all cell lines showed a similar level of high viral replication activity as well as the growth kinetics. The highest titer achieved from CCRC-1, Vero or MRC-5 following the infection with the virus of 0.1 MOI were 6.29 ± 0.19 lgCCID50/ml, 6.53 ± 0.19 lgCCID50/ml and 6.29 ± 0.19 lgCCID50/ml, respectively (Fig. 4A). However, although a similarity was seen in the viral titer, a remarkable difference in the production of viral antigen was observed among all three cell lines. The peak EV71 antigen content achieved in CCRC-1, MRC-5 and Vero following the viral infection were 789.7 ± 122.0 U/ml, 599.7 ± 49.7 U/ml and 828.7 ± 27.7 U/ml, respectively (Fig. 4B).

**Figure 5:**
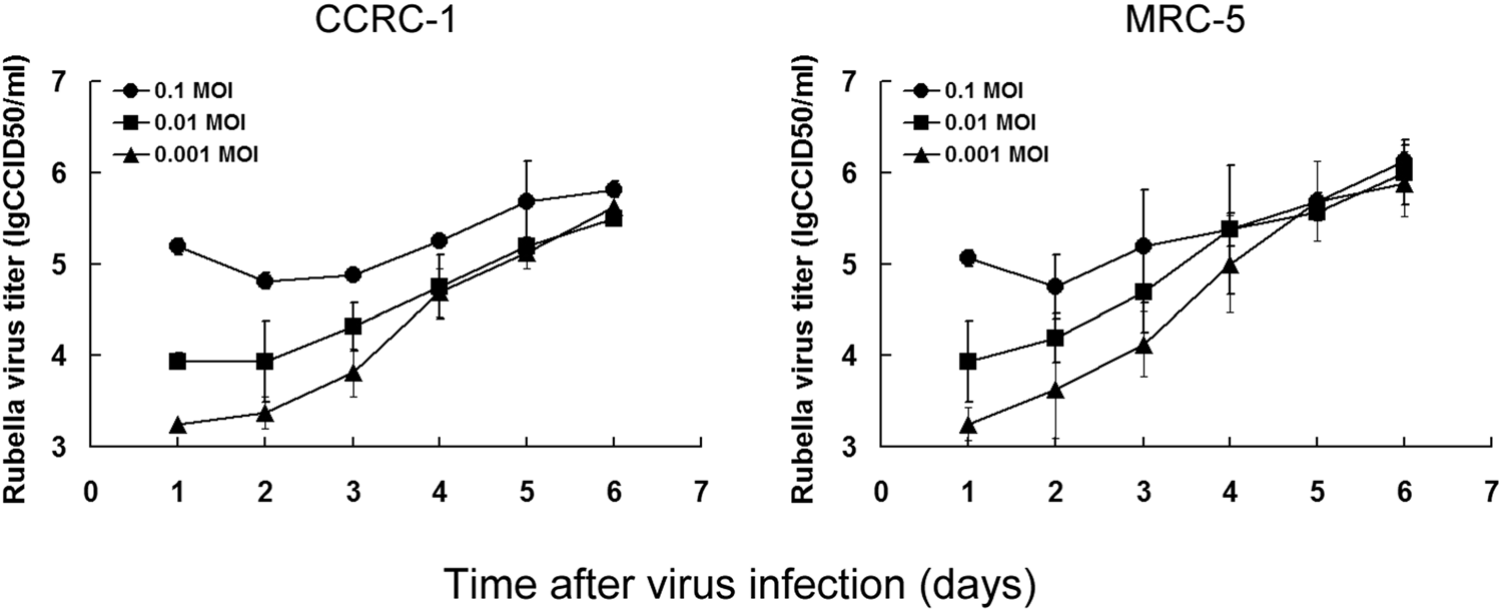
Replication activity of Rubella virus in CCRC-1 and MRC-5 cells. CCRC-1 and MRC-5 cells were infected with RUV/RA27/3 virus at MOIs of 0.1, 0.01 and 0.001, respectively. The virus titers were tested every 24 h post infection for 6 days, which were quantified using the standard CCID50 assay and expressed as lgCCID50/ml. Data are shown as the mean ± SD of three independent experiments.

Similarly, we also compared the replication activity of RUV/RA27/3, an attenuated virus strain used in the development of Rubella virus vaccine using MRC-5 cells^17^, between CCRC-1 and MRC-5 cells. It was observed from the virus replication kinetics that the virus was able to efficiently replicate in both cell lines (Fig. 5). The highest virus titers reached at day 6 in CCRC-1 cells following the infection with the virus of 0.1, 0.01 or 0.001 MOIs were 5.81 ± 0.09 lgCCID50/ml, 5.50 ± 0.02 lgCCID50/ml and 5.63 ± 0.02 lgCCID50/ml, respectively, whereas, that in MRC-5 were 6.13 ± 0.18 lgCCID50/ml, 6.00 ± 0.35 lgCCID50/ml and 5.88 ± 0.35 lgCCID50/ml, respectively.

**Figure 6:**
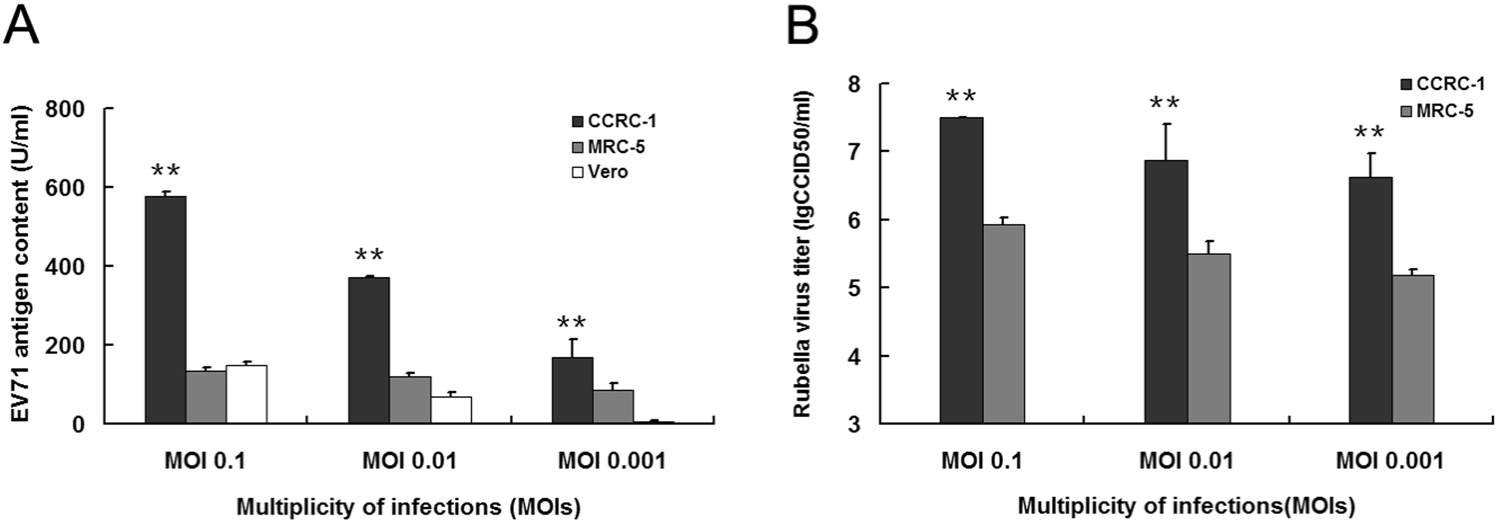
Production of EV71 and Rubella vaccine in CCRC-1, MRC-5 and Vero cells under the serum free culturing condition. The viral suspension collected from the cells, maintained in serum free MEM and infected separately with EV71/SH06 or RUV/RA27/3 virus at MOIs of 0.1, 0.01 and 0.001, were measured for either antigen content or viral titer at day 6 after virus inoculation. (**A**) EV71 antigen contents obtained in the CCRC-1 cells, MRC-5 or Vero cells. The viral antigens were detected using a quantitative ELISA assay kit and expressed as units/ml (U/ml). (**B**) The production of Rubella virus titers in CCRC-1 cells and MRC-5 cells. The viral titers were determined using the standard CCID50 assay and expressed as lgCCID50/ml. Data are represented as the mean ± SD of three independent experiments. **P < 0.01, relative to MRC-5 or Vero.

Given that the serum-free cell culture condition has been widely accepted in the production of viral vaccines especially for human use, we then determined in all three cell lines the replication activity of EV71 and Rubella viruses under the same serum-free growth condition. As a result, a significantly higher EV71 antigen content was obtained in CCRC-1 cells than in both MRC-5 and Vero cells (Fig. 6). At day 6 after infection with EV71/SH06 of 0.1, 0.01 or 0.001 MOIs, the antigen contents achieved in CCRC-1 cells were 577.0 ± 12.1 U/ml, 370.3 ± 2.8 U/ml and 169.2 ± 46.2 U/ml, respectively. However, the corresponding values obtained in MRC-5 cells were 134.3 ± 8.6 U/ml, 120.8 ± 7.5 U/ml and 85.1 ± 16.5 U/ml, respectively, and that in Vero cells were 148.1 ± 9.4 U/ml, 68.5 ± 11.8 U/ml, 5.8 ± 1.8 U/ml, respectively, thus strongly demonstrating that, under the serum-free condition, the CCRC-1 cells achieved a much higher production of EV71 vaccine than both Vero and MRC-5 cells (Fig. 6A). Similarly, under the serum-free condition, a significantly higher RUV/RA27/3 titer at day 6 was achieved in CCRC-1 cells than in MRC-5 cells (Fig. 6B). These data demonstrated that the CCRC-1 cells represent a novel high-yielding cell substrate for viral vaccine production with even a greater yield when the serum-free condition was employed.

### The EV71 vaccines derived from all three cell lines exhibited a similar immunogenicity

**Figure 7:**
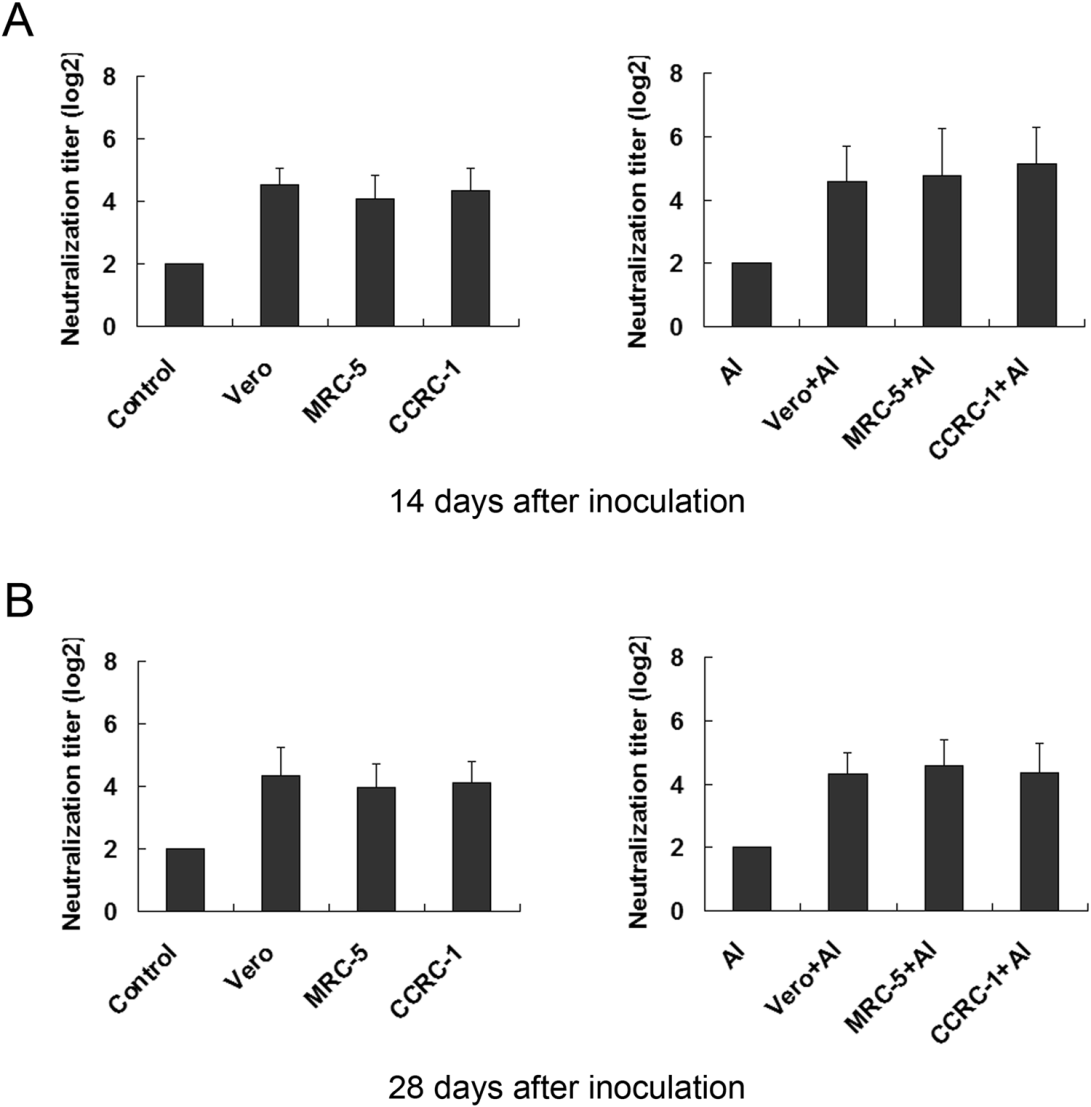
Immunogenicity of EV71 vaccines produced by CCRC-1, MRC-5 and Vero cells. The immunogenicity, as represented by the production of NAb induced in BALB/c mice by the EV71 vaccines produced from the cells with (CCRC-1+Al, MRC-5+Al and Vero+Al) or without (CCRC-1, MRC-5 and Vero) aluminum adjuvant, was detected at 14d (**A**) and 28d (**B**) after inoculation. PBS (control) and PBS plus aluminum adjuvant (Al) were used as the controls. NAb titers lower than 1:8 were assigned a value of 1:4. Common logarithmic transformation of the NAb titer raw data was used to calculate the GMT. Data are represented as the mean ± SD of the animals, n = 10.

The immunogenicity elicited by vaccines in testing animals as commonly represented by the NAbs seroconversion rate has been employed to predict clinical efficacy of the relevant vaccines^20^. To evaluate the immunogenicity, the NAbs seroconversion rates in all mice inoculated with the vaccines generated from different cell substrates were measured. Surprisingly, the immunogenicity in all vaccine-inoculated animals reached almost 100.0% at day 14 after immunization and was persistent at the same level at day 28. The Geometric Mean Titers (GMTs) of the anti-EV71 NAb elicited by CCRC-1/EV71, MRC-5/EV71, Vero/EV71, CCRC-1/EV71 + Al, MRC-5/EV71 + Al and Vero/EV71 + Al were 1:21.0, 1:19.1, 1:23.7, 1:37.6, 1:31.5, 1:26.2 and 1:18.0, 1:16.4, 1:20.6, 1:21.9, 1:25.1, 1:21.6 at day 14 and 28 post-inoculation, respectively (Fig. 7). These data indicated that, based on the induction of NAbs, the vaccines produced in CCRC-1 exhibited the same immunogenic properties as the vaccines generated in MRC-5 cells or Vero cells.

### The EV71 vaccines produced in CCRC-1 cells caused no systemic toxicity

**Figure 8:**
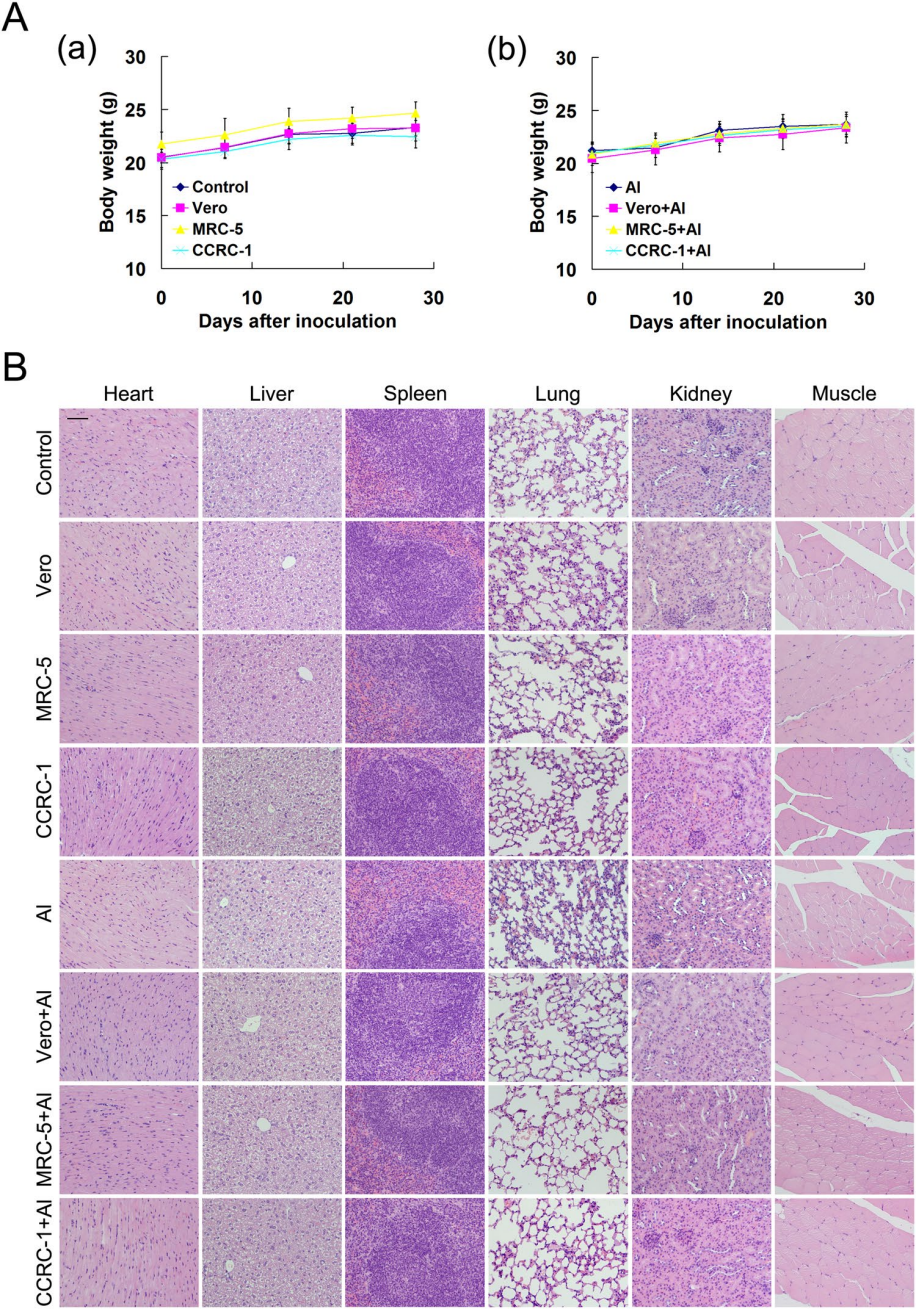
Determination of general toxicity and potential adverse effects for EV71 vaccine produced by CCRC-1, MRC-5 and Vero cells. The general toxicity and potential adverse effects EV71 vaccines produced from the cells with (CCRC-1+Al, MRC-5+Al and Vero+Al) or without (CCRC-1, MRC-5 and Vero) aluminum adjuvant were evaluated in BALB/c mice. PBS (control) and PBS plus aluminum adjuvant (Al) were used as the controls. (**A**) Weight curve of the vaccine-immunized mice, which were plotted at 3-day intervals. Data are represented as the mean ± SD of the animals, n = 10. (**B**) Histochemical analysis of tissue specimens from different groups; scale bar = 100 µm.

To evaluate the existence of any possible systemic toxicity elicited by the EV71 vaccines derived from different cell lines with or without aluminum adjuvant, the testing animals were monitored for weight change as well as other adverse findings in a three day interval over the entire observation period. The results showed that there was no significant difference in weight change among different testing groups (Fig. 8A). In addition, no other adverse findings, such as fur ruffling, abnormal behaviors, and feeding death or other unexpected death, were observed during the entire observation period. Furthermore, no pathological findings in the heart, liver, spleen, lungs, kidney or muscle were observed in all testing animals, thus suggesting that the EV71 vaccines produced from CCRC-1 cells like that from MRC-5 and Vero cells did not induce any detectable systemic toxicities (Fig. 8B).

### The hUC-MSCs lines derived from different donors exhibited an overall high efficiency in viral replication

**Figure 9:**
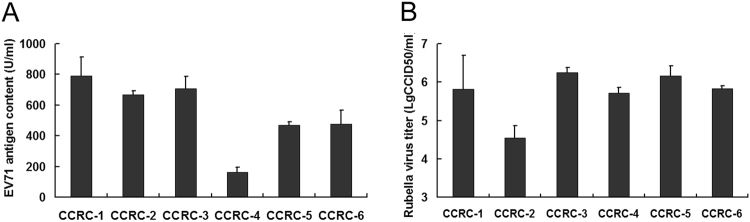
Production of viruses in different hUC-MSC strains. Antigen contents and virus titers were used to represent the production of EV71 and Rubella virus, respectively, in different hUC-MSC strains (CCRC-1, CCRC-2, CCRC-3, CCRC-4, CCRC-5 and CCRC-6). The measurements were performed at day 6 after infection with EV71/SH06 or RUV/RA27/3 at MOI of 0.1. The yields of EV71 antigen contents (**A**) or Rubella virus titers (**B**) are presented and the data are expressed as the mean ± SD of three independent experiments.

To determine the viral replication efficiency in hUC-MSCs derived from different donors, we compared the replication efficiencies of EV71-SH06 and RUV/RA27/3 in six hUC-MSC cell lines, i.e. CCRC-1, -2, -3, -4, -5, -6 lines, which were derived from 6 individual donors. The cells of each line were infected with 0.1 MOI EV71-SH06 virus or RUV/RA27/3 virus. The EV71 virus antigen content or Rubella virus titer tested at day 6 after infection was employed to represent the replication efficiency of the relevant virus. The results showed that, for EV71 virus, a relatively higher virus content was obtained in CCRC-1, -2 and -3 lines, among which the highest one was CCRC-1 (789.7 ± 122.0 U/ml). Whereas, for Rubella virus, 5 lines, i.e. CCRC-1, -3, -4, -5 and -6, produced a relatively high virus titer with CCRC-3 being the highest one (6.17 ± 0.26 lgCCID50/ml), thus demonstrating that hUC-MSCs as a group exhibited an overall high efficiency in viral replication, although the variations existed among the individual hUC-MSCs (Fig. 9).

## Discussion

Vaccines have proved to be the most efficacious and cost-effective intervention for assuring public health, and have contributed to a dramatic reduction in the spread and burden of various infectious diseases. Cell substrate is among the most critical components imposing dramatic effects on both quality and quantity of viral vaccine productions^3,4,21^. In terms of safety issues regarding tumorigenicity, HDCs represent the safest one among all types of cell substrates^1^. However, because of the limitation in propagation capacity, the continuous diminishing of early passage cells, such as the continuous reduction of the WHO MRC-5 Reference Master Cell Bank^3,4^, and the fact that all existing HDCs were derived from the fetal lung tissues, which are associated with both technical and ethical issues^17,22^, searching for new source of derivation and establishing novel HDCs is often needed. Following the previous observation^17^, we provide further evidence that the human umbilical cord rich in mesenchymal stem cells can serve as a more feasible source of HDC derivation and CCRC-1 derived from the human umbilical cord represents a novel high-yielding HDC cell line with a likely higher efficiency than the existing HDCs for the production of at least EV71 vaccines.

The quality of cell substrates is directly associated with the quality and safety of resultant products^23^. Based on major quality requirements of cell substrates, both World Health Organization (WHO) and Chinese pharmacopeia provide a set of guidelines for cell bank characterization for different cell substrates, including HDCs. The major quality requirements from the guidelines for HDCs include the freedom from adventitious agents and non-tumorigenicity^18,19^.

The contamination from adventitious agents in cell substrates represents the most important safety issue associated with cell substrate-derived vaccines^23^. It can cause extremely serious consequences not only to biopharmaceutical enterprises with huge monetary losses, but also to vaccinees with severe health losses. It can happen via a variety of routes including previously undetected contamination of cell substrates themselves, the route from starting materials or from improper operations^23^. In the present studies, we demonstrated clearly that the established CCRC-1 banks were free from the contamination of adventitious agents, thus suggesting that they fully meet the requirements of cell substrate for production of viral vaccines.

The main concerns associated with tumorigenicity of cell substrates can be addressed by testing their potential formation of tumor allografts in immuno-compromised animals, or by testing the potential oncogenicity derived from likely oncogenic components of cell substrates that might induce tumor formation from the cells of testing animals^24,25^. It is also important to notice that tumorigenicity among different cell strains or different passage levels of the same strain can be significantly different. Although recent evidence demonstrated that the prolonged *in vitro* proliferation may cause genomic mutations in hMSCs, the tumorigenic transformation was extremely rare for hMSCs within the limited passage levels (at least less than 60–100 passages)^26,27^. Here, we found that CCRC-1 cells at all passage levels tested did not form tumor in nude mice, and maintained an extremely low hTERT activity (which serves as a surrogate biomarker of tumorigenicity, Table 2 and Fig. 1), these results suggest that tumorigenicity should not be a safety concern for CCRC-1 cells within limited passages.

It is ideal that the cells used for vaccine production should grow fast and can reach to maximal density to accommodate maximal viral replication^28^. Although most HDCs at low passage levels may grow fast, they may increasingly lose their growth activity when reaching to high passage levels^29^. Interestingly, the observations in this study demonstrated that, different from most hHDCs and superior to MRC-5, the CCRC-1 cells at high passage levels, i.e. passage 30, can still keep a similar growth rate and reach to the density pattern as low-passage cells (Fig. 2), thus suggesting that, in terms of the growth density feature, the CCRC-1 cell line is superior to MRC-5 line for vaccine production, which may be beneficial for shortening the production cycles and improving the yield of viral vaccines.

In order to meet the needs of producing viral vaccines of large variety, the cell substrate should have a broad spectrum of viral susceptibility. The high susceptibility to the infection of a vast variety viruses is shared by the most frequently used cell substrates, such as MRC-5 cells and Vero cells^30–33^, thus should serve as a prerequisite feature for novel cell substrates. In this study, we revealed that CCRC-1 shares a similarly high susceptibility to the infection of various viruses with MRC-5. In addition, as another important necessity for virus replication, the cell substrate has to be competent for achieving high yield viral production^34^. Consistent with the finding revealed in the previous study^17^, we found that both EV71/SH06 and RUV/RA27/3 could replicate to high levels in CCRC-1 cells. Importantly, under the serum-free culturing condition, the production of both EV71 and Rubella vaccines in CCRC-1 cells was significantly higher than that in MRC-5 or Vero cells. This high compatibility to viral replication is believed to be attributable to the unique stemness of CCRC-1 cells, which probably makes the cells surviving longer in serum-free culturing condition^12,13^. This finding is highly relevant to the future vaccine production as the serum-free culturing conditions can better meet the industry needs and regulatory requirements for human viral vaccine production than the serum-containing conditions.

To further meet the regulatory requirements, both immunogenicity and safety need to be carefully evaluated for approval of new viral vaccines as the immunogenicity of viral vaccines may be altered by post-translational modifications in host cells^35,36^. The present study showed that all the EV71 vaccines produced from CCRC-1, MRC-5 and Vero cells with or without adjuvant were able to boost a significant antibody response in 100% of the immunized mice without any detectable adverse events, suggesting that the vaccines produced by CCRC-1 cells exhibited a comparably high immunogenicity and general safety. Nevertheless, more studies are warranted for further investigating the efficacy and safety of the viral vaccines produced by CCRC-1 cells before their clinical applications

Given that, as a novel cell substrate for viral vaccine production, CCRC-1 is superior to both MRC-5 and Vero as revealed in this study, the superiority may represent a common feature of hUC-MSCs, which may be superior as a group to the traditional cell substrates. Indeed, we further revealed that all six hUC-MSC lines tested in this study exhibited a high yield in the production of either EV71 or Rubella virus. In the meantime, the tested hUC-MSCs also exists a clear variation regarding the ability to support the infection of different viruses. This variation in virus specificity may reflect genetic heterogenicity of each individual hUC-MSCs, which is probably associated with the difference in post-translational modification of key proteins involved in the production of each specific virus. Based on this finding, it is encouraged in the future to generate a library of different hUC-MSC lines derived from different donors with each individual line chosen for production of each specific viral vaccine.

In addition, before further developing CCRC-1 as a novel cell substrate for vaccine industry, several other issues are needed to be addressed. For example, the potential for scale-up vaccine production needs to be tested as all investigations regarding CCRC-1 revealed in this study and the previous one were performed in the size-limited cell culture systems. In the future, different scale-up systems, such as Cell Factories or Bioreactor, will be tested for further demonstrating the superiority of CCRC-1 cells over other cell substrates. The efficiency issue regarding the downstream vaccine purification process should also be tested in comparison with other cell substrates. Moreover, in addition to the EV71, which has proved in this study to be suitable for using CCRC-1 as cell substrate in vaccine production, the suitability for other viruses may hold an equal promise and warrants further investigation in the near future.

In conclusion, we further prove that the CCRC-1 cell line, which may represent a novel category of HDCs, is significantly superior to the traditional cell substrates in safety, cell derivation and viral production. Given the continuous diminishing of the existing HDCs and difficulties in deriving new HDCs from human fetal lung tissues, the findings revealed in this study is very important because it provides a fundamental and efficient solution to solve the problems associated with the existing HDCs. More realistically, through this study, a three-tiered CCRC-1 bank system has been successfully established and fully characterized according to the industry standards and may be served as a more efficient HDC line than MRC-5 at least in the production of EV71 and Rubella viral vaccines. With the existing evidence and new upcoming investigations, the potential value of CCRC-1 in viral vaccine industry may be realized in the near future.
